# Individual variation underlies large‐scale patterns: Host conditions and behavior affect parasitism

**DOI:** 10.1002/ecy.4478

**Published:** 2024-12-09

**Authors:** Allison M. Brehm, Vania R. Assis, Lynn B. Martin, John L. Orrock

**Affiliations:** ^1^ Department of Integrative Biology University of Wisconsin‐Madison Madison Wisconsin USA; ^2^ Global and Planetary Health University of South Florida Tampa Florida USA

**Keywords:** ectoparasite, host–vector interactions, *Peromyscus*, zoonoses

## Abstract

Identifying the factors that affect host–parasite interactions is essential for understanding the ecology and dynamics of vector‐borne diseases and may be an important component of predicting human disease risk. Characteristics of hosts themselves (e.g., body condition, host behavior, immune defenses) may affect the likelihood of parasitism. However, despite highly variable rates of parasitism and infection in wild populations, identifying widespread links between individual characteristics and heterogeneity in parasite acquisition has proven challenging because many zoonoses exist over wide geographic extents and exhibit both spatial and temporal heterogeneity in prevalence and individual and population‐level effects. Using seven years of data collected by the National Ecological Observatory Network (NEON), we examined relationships among individual host condition, behavior, and parasitism by *Ixodid* ticks in a keystone host species, the white‐footed mouse, *Peromyscus leucopus*. We found that individual condition, specifically sex, body mass, and reproductive condition, had both direct and indirect effects on parasitism by ticks, but the nature of these effects differed for parasitism by larval versus nymphal ticks. We also found that condition differences influenced rodent behavior, and behavior directly affected the rates of parasitism, with individual mice that moved farther being more likely to carry ticks. This study illustrates how individual‐level data can be examined using large‐scale datasets to draw inference and uncover broad patterns in host–parasite encounters at unprecedented spatial scales. Our results suggest that intraspecific variation in the movement ecology of hosts may affect host–parasite encounter rates and, ultimately, alter zoonotic disease risk through anthropogenic modifications and natural environmental conditions that alter host space use.

## INTRODUCTION

A comprehensive understanding of the mechanisms underlying host–vector interactions is crucial to improve human health globally, as many of the pathogens that cause human disease are zoonotic pathogens that infect wildlife hosts (Jones et al., 2008). These pathogens are often vectored by arthropods (e.g., mosquitos, ticks, fleas) that parasitize vertebrate hosts. Indeed, fundamental to the challenge of understanding the dynamics of zoonotic diseases is that parasites are frequently highly aggregated among individual hosts (Poulin, 2007), often leading to extreme variation in parasite prevalence in time and space. Such variation is observed at the intraspecific and interspecific levels (i.e., superspreader individuals or diluter species) (Martin et al., 2019), and may be spatiotemporally variable (i.e., hotspots of pathogen prevalence) (Paull et al., 2012). To grapple with such extreme heterogeneity in systems often characterized by multiple hosts spread over large spatial scales, we must find a way to understand the causes of variation in key components of zoonoses, such as host–parasite encounters.

Particular behaviors may provide insight into variation in host–vector interactions at multiple levels of organization (Sih et al., 2018). For instance, individuals that are more central in social networks or are highly mobile may more often contact vectors and other hosts, making them more likely to be superspreaders (Martin et al., 2019; Paull et al., 2012). Similarly, species that are highly social and, thus, readily acquire and transmit parasites, may be termed “amplification hosts” (Paull et al., 2012). By contrast, species that are highly effective at killing parasites may be “superdiluters” (Martin et al., 2019). Hotspots may arise spatially at feeding sites, breeding grounds, or watering holes, or temporally due to seasonal shifts in behavior (i.e., migration, breeding, wintering grounds) (Paull et al., 2012). To determine how and when behavior underpins extreme variation in host–parasite contact, it is important to appreciate that intrinsic and extrinsic factors, too, can alter behavioral variation. In other words, consequential behavioral variation can arise due to individual condition differences (i.e., sex, age, body size, or reproductive status), consistent among individual differences (i.e., personality, genetic/epigenetic factors) (Sih et al., 2004), or spatial or temporal heterogeneity in the environment (Guiden & Orrock, 2017; Lima & Dill, 1990). An additional complexity is that intrinsic and extrinsic factors may interact; some metrics of individual condition may depend on the environment. As a result, studies capable of probing how behavioral variation arises within the biotic and abiotic milieu that characterizes ecological communities may be particularly useful for understanding zoonotic disease prevalence.

Rodents comprise a disproportionate number of zoonotic disease reservoirs (Han et al., 2015), and many rodent species are key to maintaining enzootic cycles for many tick‐borne diseases. In North America, among the most common and consequential are Lyme disease, human granulocytic anaplasmosis, babesiosis, ehrlichiosis, tick‐borne relapsing fever, and Powassan viral encephalitis (Barbour, 2017; Larson et al., 2021). These tick‐borne diseases are vectored by a family of ectoparasites known as *Ixodid* ticks, or the hard‐bodied ticks, which include the blacklegged tick, *Ixodes scapularis*, the lone star tick, *Amblyomma americanum*, and the dog tick, *Dermacentor variabilis*, among others (Nieto et al., 2018; Tsao et al., 2021). Encounters between rodents and *Ixodid* ticks are fundamental to cycles of tick‐borne diseases because many of these diseases lack vertical transmission. As such, larval ticks are typically infected only when feeding on an infected rodent, and rodents become infected only when they are parasitized by an infected tick. Heterogeneity in tick burdens among individual rodent hosts depends on various morphological and ecological attributes of individuals (Devevey & Brisson, 2012). For instance, males are generally more heavily parasitized than females, and parasitism is generally higher in larger‐bodied individuals (Brunner & Ostfeld, 2008; Devevey & Brisson, 2012; Gorrell & Schulte‐Hostedde, 2008; Larson et al., 2018; Moore & Wilson, 2002; Perez‐Orella & Schulte‐Hostedde, 2005). Ultimately, however, parasite acquisition depends upon the parasite and host being present in the same place at the same time. Thus, the magnitude, location, and timing of host movements should also strongly influence host–parasite contact rates (Sih et al., 2018). Indeed, individuals that are more trappable (i.e., potentially bolder or ranging individuals), are more active or exploratory, or more often share the same refuges as others have been shown to carry greater parasite burdens (Boyer et al., 2010; Hall, 2022; Wilder & Meikle, 2004). Associations between host space‐use and parasite acquisition may be particularly pronounced regarding contact with juvenile *Ixodid* ticks because they are aggregated in space and quest for hosts using an ambush strategy (Leal et al., 2020; Ostfeld, Hazler, & Cepeda, 1996) increasing the probability that individuals with greater movements or larger home ranges will make contact (Brunner & Ostfeld, 2008).

The white‐footed mouse (*Peromyscus leucopus*) is among the most abundant and geographically widespread rodents in North America. As a primary host for the larval stage of *Ixodid* ticks, such as the blacklegged tick (*I. scapularis*), *P. leucopus* is a reservoir for numerous tickborne pathogens (Larson et al., 2018), and is the main reservoir for the pathogen that causes Lyme disease, the spirochete bacterium *Borrelia burgdorferi* (Kugeler et al., 2021; Lane et al., 1991; Schwartz et al., 2021). Lyme disease is a public health concern throughout the eastern and Midwestern US, where it is estimated that 476,000 are diagnosed and treated each year (Hahn et al., 2016; Kugeler et al., 2021; Schwartz et al., 2021), and up to 95% of blacklegged ticks feeding on *P. leucopus* become infected with *B. burgdorferi* (Ostfeld, 2011). *P. leucopus* is, therefore, an appropriate target species in which to probe for generalizable trends between individual host traits and parasite encounters. Despite the extensive nature of *B. burgdorferi* and other zoonotic infections in mice and humans, as well as the importance of rodent densities in maintaining disease prevalence and human disease risk (Ostfeld et al., 2006), we still know comparatively little about the widespread roles of host conditions and behavior in affecting parasitism, and whether these are geographically consistent over the host's range. One possibility is that generalizations remain elusive because of the logistical difficulty of conducting studies to understand links between behavior and parasitism across the large spatial and temporal scales that characterize the dynamics of widespread zoonoses. Indeed, these logistical difficulties are reflected in the relatively local scales at which aforementioned studies examining ecological correlates of ectoparasite prevalence/burdens were performed. The recent creation of large‐scale vertebrate monitoring networks, such as the National Ecological Observatory Network (NEON) provides an unprecedented opportunity to evaluate the ecological consequences of animal behavior (Brehm & Orrock, 2023) at broad geographic scales.

Using multi‐site, multi‐year data from NEON, we examined relationships among individual conditions, behavior, and presence of an ectoparasite. Via structural equation modeling (SEM), we disentangled direct and indirect relationships among condition traits (e.g., sex, body mass, reproductive state), behaviors (e.g., movement distance, trappability, trap diversity), environmental context (forest type), and ectoparasite attachment (e.g., occurrence of parasitism by juvenile *Ixodid* ticks) in *P. leucopus*. We expected that individuals that were more trappable, used more trap locations, and moved greater distances between repeated trapping events would be more likely to be parasitized by ticks because greater levels of movement activity would be expected to increase encounter rates with questing ticks. Alternatively, more movement may result in less grooming behavior due to a limited time budget, potentially also leading to increased parasitism by ticks (Hart & Hart, 2018). We also expected that reproductively‐capable males would be most likely to be parasitized by ticks, as these individuals might have the weakest immune defenses (Hughes & Randolph, 2001). This study should clarify whether such generally accepted patterns as sex‐biased parasitism hold up across years, geographic regions, and forest types, where there may be pronounced variation in the abundance and activity patterns of rodents and ticks (Ostfeld, Hazler, & Cepeda, 1996). Moreover, this work should elucidate general relationships pertaining to individual correlates of zoonotic disease transmission and the capacity for behavioral variation to generate heterogeneity in pathogen/parasite prevalence in time and space.

## METHODS

### Dataset assembly

#### Small mammal trapping data

We examined small mammal live‐trapping records from NEON from 2013 through 2022 (NEON Doc. number: NEON.DOC.000481). Briefly, NEON traps small mammals using 100 Sherman live traps (H. B. Sherman, Inc., Tallahassee, FL, folding or non‐folding, 3″ × 3.5″ × 9″) laid out in a trapping grid with 10 m spacing. Traps are baited with a mixture of sunflower seeds and millet for three consecutive nights within each sampling period. Sampling timing varies depending upon whether sites are deemed core or gradient, but sampling at sites used in this study was performed most commonly on a monthly or bimonthly basis between the months of April and October. Upon capture, individual small mammals are marked with a unique tag and identified to species if possible. Morphology is assessed for age, sex, and reproductive condition, and standard measurements are taken (such as hind foot length and body mass).

To ensure that our findings were relevant for the most prevalent zoonosis in North America, Lyme disease, we filtered observations to NEON sites within the estimated and established range of *I. scapularis* (https://www.cdc.gov/ticks/geographic_distribution.html). We included only data from mammal trapping grids located within forested habitats (obtained using the “nlcdClass” identifier in the mammal trapping dataset), extracted only capture events, and removed observations where the trap location was unknown or the individual was not tagged (e.g., escapes or trap deaths). Since distinguishing between *P. leucopus* and the closely related woodland deer mouse, *P. maniculatus*, is difficult in the field (Stephens et al., 2014), we initially retained all captures identified in the field as either of the two species. We limited observations to individuals of known sex, and we excluded juveniles (juveniles constituted only ~3% of captures in the NEON rodent‐trapping data after all other data filtering had been performed). Then, we performed a three‐step process to retain only individuals with a confident *P. leucopus* taxonomic identification (Appendix S1: Figures S1–S9, Boxes S1 and S2). Though both *Peromyscus* species may act as hosts of *I. scapularis* and other *Ixodid* ticks (Larson et al., 2018, 2021; Yen et al., 2024), we chose to focus on *P. leucopus* due to their wider geographic distribution across NEON sites.

#### Ectoparasite data

NEON added tick monitoring to the small mammal trapping protocol in 2016, classifying ticks attached to the face and ears of small mammals by life stage (i.e., larval, nymphal, or adult ticks), as these are the primary locations where juvenile *Ixodes* ticks are observed attached to mice (Main et al., 1982; Ostfeld et al., 1993). NEON began estimating total tick count binned estimates in 2020, but in this study, we refer to tick presence/absence on small mammals only. Attached ticks are not identified to species in the field, but NEON also samples tick abundance and diversity using drag or flag sampling techniques at regular intervals at core and relocatable sites (NEON Doc number: NEON.DOC.014045). From drags performed in forested habitat (deciduous forest, evergreen forest, mixed forest, or woody wetlands) at the 16 NEON sites we use in this study, a total of 472,832 ticks were collected and identified to family and, if possible, species. Of these, 100% belonged to the family *Ixodidae*, or hard‐bodied ticks. Over 95% were identified to the family of hard ticks only (*Ixodidae* sp.). Of the ticks identified to species, these were primarily identified to the genus *Amblyomma* (primarily *A. americanum*; 73%) and *Ixodes* (primarily *I. scapularis*; 23%) and <1% were identified as *D. variabilis*.

#### Individual movements, trappability, and trap‐use diversity

For all individual mice with two or more trapping observations, we estimated the distance in meters between all consecutive trapping locations using the R package “geosphere” (Hijmans et al., 2022). We interpret longer movement distances between captures as being indicative of greater activity. Because mice were sampled during multiple three‐day sessions, a movement event could occur within a session or between a session. In our analyses, we did not distinguish between the two different types of movement (within‐session vs. between‐session movement) because the type of movement explained less than 0.01% of the variation in movement distance (see Appendix S2: Table S1 and Figure S1).

Trappability refers to the tendency of an individual to be captured and, here, is used synonymously with individual capture probability. Trappability is sometimes interpreted as a measure of boldness (Boyer et al., 2010; Carter et al., 2012; Patterson & Schulte‐Hostedde, 2011; Santicchia et al., 2018), but may also signify food motivation and whether an individual becomes “trap happy” or “trap shy” after an initial capture event (Nichols et al., 1984); indicating the tendency to alter behavior in response to cost/benefit information. We calculated trappability by dividing an individual's total number of capture events by the total number of trap nights that occurred during an individual's residence time (i.e., between and including the first and last observation of an individual). Trappability scores are bounded between zero and one. Estimates closer to one represent high trappability, while estimates closer to zero represent low trappability.

For each mouse, trap‐use diversity was calculated as the number of different trap locations used divided by the total number of capture events. Trap‐use diversity is used as a measure of an individual's exploratory tendency (Boyer et al., 2010), and estimates are bound between zero and one. Estimates closer to zero represent a tendency to utilize the same trap upon subsequent captures, while estimates closer to one represent a tendency to utilize a different trap upon subsequent captures. A higher trap‐use diversity (hereafter referred to as trap diversity) score may be interpreted as evidence of more exploratory movement behavior.

The three behavioral metrics obtained from the rodent‐trapping data encapsulate among individual differences in space use, such as whether individual space‐use can be described as more “ranging” or “restrained”. However, we acknowledge that qualities of the individual and features of the environment will directly affect these measures. For example, subadult individuals may exhibit increased activity and space use during natal dispersal (Hoset et al., 2011), males often have larger home ranges than females and generally move longer distances than females (Ostfeld, Miller, & Hazler, 1996). Further, home range size (an indicator of movement) may be predicted by rodent population density, and home ranges are generally smaller in years of high rodent density (Wolff, 1985) or increased resource availability (Stradiotto et al., 2009; Wolff, 1985). The interconnectedness of multiple traits used in this study necessitates the use of analytical methods that can deal with such ecological complexity (Fan et al., 2016).

### Structural equation models

We used piecewise SEM to identify the causal effects (i.e., direct and indirect relationships) among morphology, forest type, activity levels, exploratory behavior, and ectoparasite attachment. SEM is a form of path analysis that defines complex statistical relationships among nodes in a network (variables) connected through paths (Lefcheck, 2016; Shipley, 2002). In an SEM, variables can be defined as either predictor, response, or both. Piecewise SEM is a recent development, and by using local estimation to allow the implementation of random effects, different correlation structures, and non‐normal distributions, these models offer greater statistical flexibility than traditional SEMs (Lefcheck, 2016). For these reasons, they are useful in ecological analyses where relationships are complex and often suites of variables with different error distributions are interrelated (Fan et al., 2016).

**FIGURE 1:**
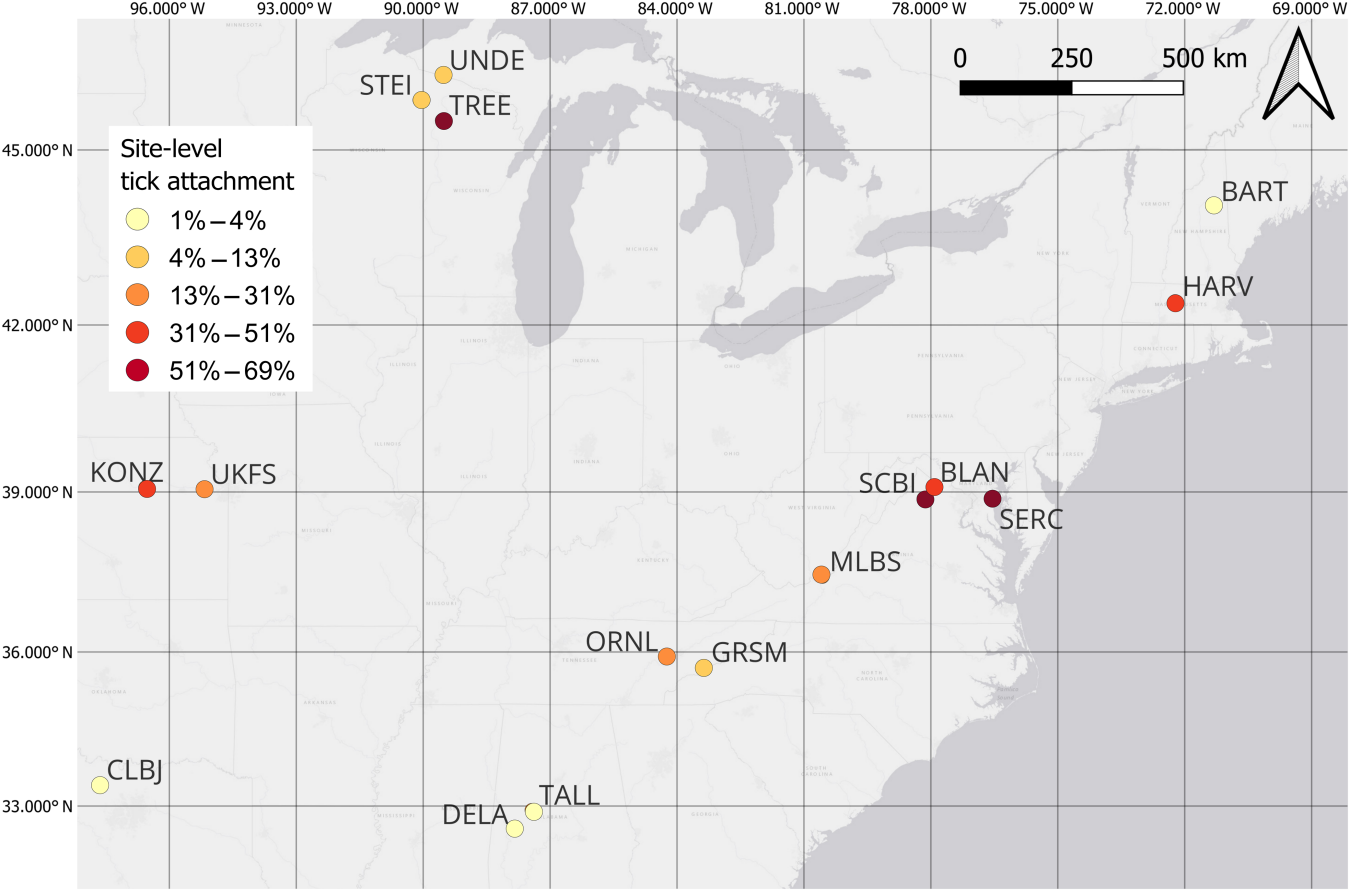
Geographic extent of the National Ecological Observatory Network (NEON)'s small mammal trapping data used in this study. Each circle represents one core NEON site. Site‐level tick attachment is indicated in the figure legend (defined as the percentage of white‐footed mouse, *Peromyscus leucopus*, captures with one or more ticks attached).

We used piecewise SEMs structured using the “piecewiseSEM” package in program R (Lefcheck, 2016). Our dataset contained some variables with repeated measures (i.e., body mass, movement distance, tick attachment) and other variables that were invariant within an individual (such as sex, trappability, plotID, and trap diversity). To avoid pseudoreplication, we used just one observation per individual and summarized each repeated measure in the following ways: we used each individual's mean values for body mass and movement distance, created a variable representing reproductive tendency (i.e., the proportion of captures when the individual was observed in a reproductive state), and estimated individual occurrence of parasitism by a tick (i.e., the proportion of captures when the individual had a tick attached to the face or ears) at two tick life stages: larval and nymphal. We extracted all complete observations for the following variables (sex, average body mass, average movement distance, proportion of captures reproductive, occurrence of parasitism by larval ticks, occurrence of parasitism by nymphal ticks, trappability, and trap diversity). For average body mass only, we replaced missing values with the mean value for the species (~1% of observations). The final dataset used for SEM analyses contained 3956 individual *P. leucopus* from 81 different trapping grids across 16 NEON sites around the Midwestern and eastern US (Figure 1). The distribution of individuals across forest types was as follows: 3253 individuals were present in trapping grids in deciduous forest, 412 in evergreen forest grids, 254 in mixed forest, and 37 in woody wetlands.

We created an SEM (base model) comprised of 5 linear mixed models (with plotID as a random effect in each to acknowledge the lack of independence of captures from the same site, and account for spatial heterogeneity in tick abundance) (Larson et al., 2021): (1) Movement distance as the response variable, (2) trappability as the response variable, and (3) trap diversity as the response variable. In each, condition variables suspected to influence behavior were specified as fixed effects (i.e., average body mass, sex, and proportion of captures reproductive). The last two models specified (4) parasitism by larval ticks, and (5) parasitism by nymphal ticks as the response variables, and forest type as a fixed effect. Due to the uneven nature of these data among the forest types, we classified grids as either deciduous (3253 individuals) or not (703). We chose to use forest type rather than other environmental parameters, because many parameters of interest (i.e., precipitation or temperature during the trapping period) would have been repeated measures, and much of the variance in habitat structure or climate differences between sites is explained by the random effect of plotID. We used tests of directed separation to assess each claim of independence in the model, and restructured the model adding fixed effects if the test of directed separation and Fisher's C statistic indicated a missing link was present (Lefcheck, 2016; Shipley, 2002). In this restructured model we also removed any nonsignificant fixed effects from the base SEM so as not to overparameterize the model. Lastly, in the final, restructured SEM, we tested five different interactions specifying parasitism by ticks: (sex × proportion of captures reproductive), (sex × average body mass), (sex × movement distance), (proportion of captures reproductive × movement distance), and (forest type × movement distance). We tested each interaction in a separate competing model. We compared these models with the restructured SEM without interactions, and if an interaction term was significant and the model was better supported (by Akaike's information criterion corrected for small sample sizes using a threshold of 2.0 ΔAIC_c_; Burnham & Anderson, 2002), we considered this to be the final, best supported SEM. This SEM structure allowed us to (1) examine the effects of intraspecific behavioral differences on parasitism by ticks, (2) assess whether individual conditions had a direct effect on parasitism or acted indirectly through behavior, and (3) compare the morphological and behavioral correlates of tick parasitism in *P. leucopus*. All continuous variables were z‐scaled and proportional response variables (i.e., trappability, trap diversity, and parasitism by ticks) were logit‐transformed (Warton & Hui, 2011). See Appendix S3: Figure S1 for the original metamodel structure before paths were added based on suggestions from modification indices.

## RESULTS

**FIGURE 2:**
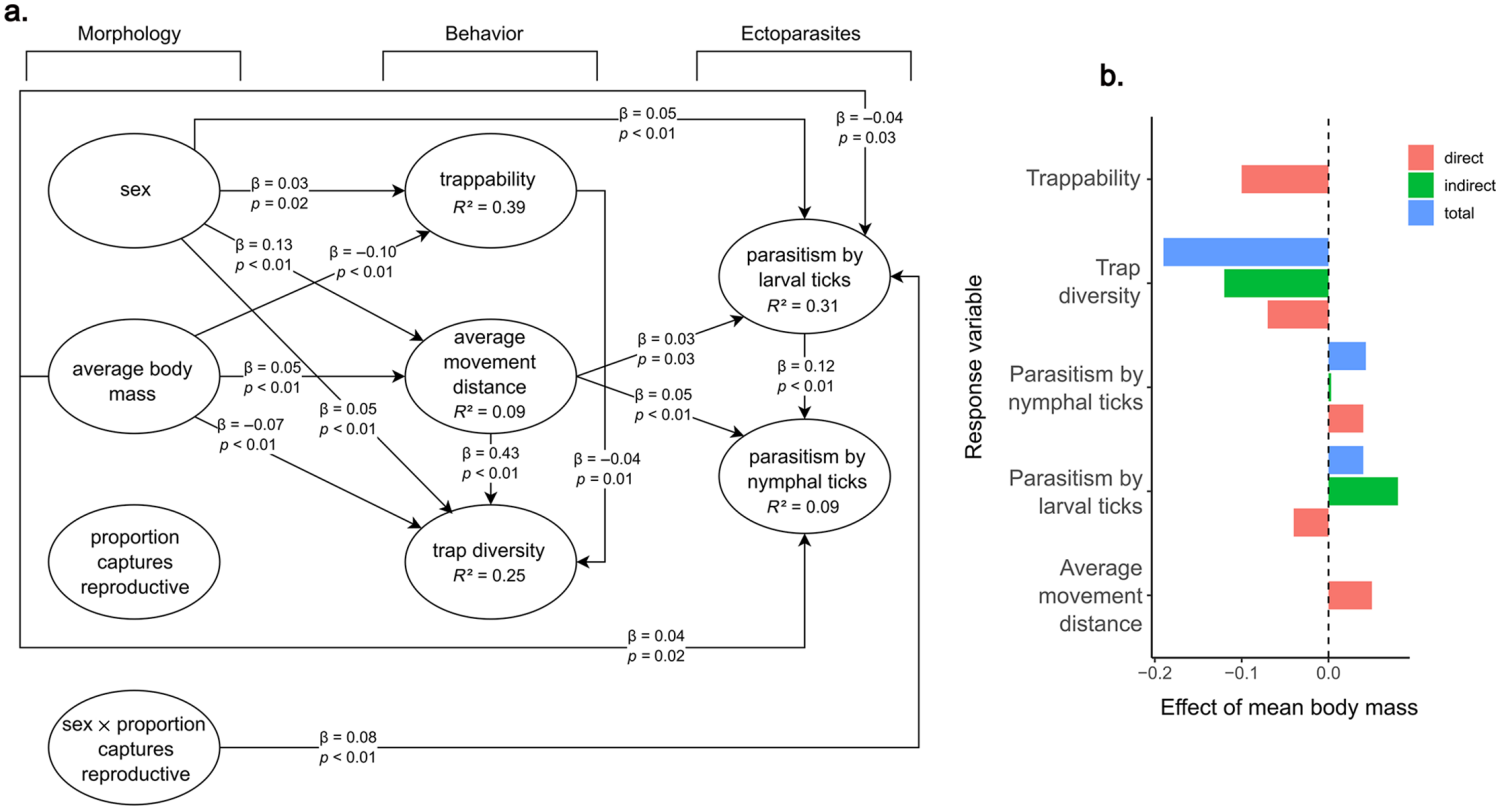
(a) Structural equation models showing direct and indirect relationships between individual conditions, behavior, and parasitism by ticks in the white‐footed mouse, *Peromyscus leucopus*. β values are the standardized effects sizes and represent the strength of relationships between the variables. As continuous variables were scaled prior to analyses, these effects sizes can be directly compared. *R* ^2^ values shown are the conditional *R* ^2^ for that response variable (conditional on both fixed effects and the random intercept of trapping grid ID). Marginal *R* ^2^ values for trappability, average movement distance, trap diversity, parasitism by larval ticks, and parasitism by nymphal ticks are 0.01, 0.02, 0.19, 0.01, and 0.02, respectively. (b) Direct, indirect, and total effects of average body mass on behavior (trappability, trap diversity, average movement distance), and the occurrence of parasitism by larval and nymphal ticks.

We examined 3956 mice collected from 81 different trapping grids across 16 NEON sites during 951 unique sampling sessions, spanning a range of seven years (Figure 1). Tick parasitism was prevalent in *P. leucopus* (36% of individuals observed with a tick attached at least once) and was higher for larval ticks than nymphal ticks (31% vs. 9%, respectively); 34% of individuals were observed with only larval ticks attached, 4.8% with only nymphs attached, and 16% with both life stages attached. Sex ratios of captures were male‐biased (56% males). For a full summary of the variables used in the SEM structures, see Appendix S4: Table S1. For path diagrams of all effect sizes, *p*‐values, and *R* ^2^ values, see Figure 2.

### Behavior

Males moved farther between consecutive captures than females (Appendix S5: Figure S1a; direct β = 0.13, *p* < 0.01), and heavier individuals also moved farther distances between consecutive captures than lighter ones (Appendix S5: Figure S1a; direct β = 0.05, *p* < 0.01). Males had higher trappability than females (Appendix S5: Figure S1b; direct β = 0.03, *p* = 0.02), but heavy individuals were less likely to be repeatedly trapped than light ones (Appendix S5: Figure S1b; direct β = −0.10, *p* < 0.01). Males had greater trap diversity than females (Appendix S5: Figure S1c; direct β = 0.05, *p* < 0.01, indirect β = 0.05, total effect = 0.10), but individuals with greater average body mass had lower trap diversity (Appendix S5: Figure S1c; direct β = −0.07, *p* < 0.01, indirect β = −0.12, total effect = −0.19). Trap diversity was positively associated with average movement distance between consecutive captures (direct β = 0.43, *p* < 0.01) and negatively associated with individual mouse trappability (direct β = −0.04, *p* = 0.01).

### Tick occurrence on mice

**FIGURE 3:**
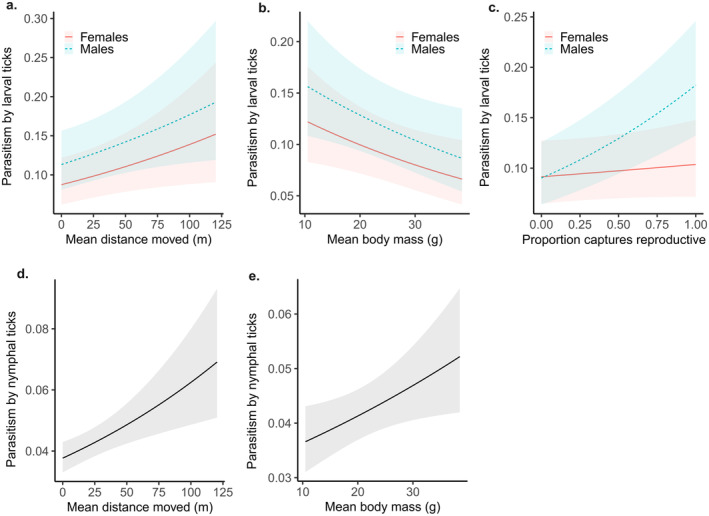
Predicted relationship (and 95% CI) between individual behavior, individual conditions, and parasitism by ticks (proportion of captures when an individual was observed with one or more ticks attached to the face or ears) in the white‐footed mouse, *Peromyscus leucopus*. (a–c) Male mice have greater occurrence of parasitism by larval ticks than females. (a, d) Mice that move farther on average have a greater occurrence of parasitism by larval and nymphal ticks. (b, e) Larger‐bodied individuals have a lower occurrence of parasitism by larval ticks but greater parasitism by nymphal ticks. (c) Male mice that are more often observed in a reproductive state have greater parasitism by larval ticks, but there is no relationship in females. Mean movement distance is in meters and mean body mass is in grams. Parasitism by larval and nymphal ticks has been back‐transformed from a logit‐transformation.

Individual mice that moved greater distances between consecutive captures were more likely to carry a larval tick (Figure 3a; direct β = 0.03, *p* = 0.03), but individuals with greater body mass had a lower occurrence of parasitism by larval ticks (Figure 3b; direct β = −0.04, *p* = 0.03, indirect β = 0.08, total effect = 0.04). Males that were more often observed in a reproductive state were more likely to have a larval tick, but this effect was absent for females (Figure 3c; direct β = 0.08, *p* < 0.01), and males had a higher overall occurrence of parasitism by larval ticks than females (Figure 3; direct β = 0.05, *p* < 0.01, indirect β = 0.004, total effect = 0.054). Individuals with greater body mass were more likely to carry a nymphal tick (Figure 3d; direct β = 0.04, *p* = 0.02, indirect β = 0.003, total effect = 0.043), and individuals that moved farther between consecutive captures were more likely to carry a nymphal tick (Figure 3e; direct β = 0.05, *p* < 0.01). Parasitism by larval ticks was also positively associated with parasitism by nymphal ticks (direct β = 0.12, *p* < 0.01). All reported β terms are the standardized estimates of effect size. As continuous variables were z‐scaled prior to analyses, the associated β terms represent the strength of relationships between the variables and can be directly compared. Therefore, the strongest direct effect on occurrence of larval tick infestation prevalence was the interaction term of sex × reproductive condition, and the strongest direct effect on occurrence of nymphal tick infestation prevalence was the prevalence of infestation by larval ticks.

## DISCUSSION

A primary challenge in understanding the dynamics of vector‐borne zoonotic diseases is finding a means to predict the encounters among hosts and vectors that determine pathogen transmission. This endeavor is complicated by the nature of zoonoses themselves, as the zoonotic diseases that affect humans, such as *B. burgdorferi* (the agent causing Lyme disease), play out in systems spanning large geographic expanses with the various important forces shaping disease dynamics manifesting over weeks, months, and even years. Using an unprecedented seven‐year dataset spanning the eastern and Midwestern US, we found that individual host behavior and conditions are key to understanding the likelihood of encounters between *P. leucopus* and ticks carrying important zoonotic agents. Our results have several important implications: First, they illustrate how large‐scale datasets can reveal broad patterns about the roles of individual host conditions and behavior in host–vector interactions across large spatial and temporal scales. Second, by showing a clear role for individual movement in the occurrence of parasitism, they indicate how factors that affect individual space use (e.g., resource availability) can have important, often unappreciated influences on pathogen transmission (Stradiotto et al., 2009; Svoboda et al., 2019; Wolff, 1985). Third, they highlight the importance of local context as a driver of host–parasite encounters. Below, we discuss our results considering these implications and explore future directions.

### Host conditions and behavior predict parasitism by ticks across large geographic areas

Given the repeated observation of high levels of variation in parasitism among hosts, there is a pressing need to understand why all hosts are clearly not equal in their likelihood of being parasitized. We found that individual host conditions, specifically sex, body mass, and reproductive condition, had both direct and indirect effects on the occurrence of parasitism by ticks, but the nature of these effects differed depending on tick life stage. Sex was an important predictor of parasitism by larval ticks: males were more likely to be parasitized than females, and males also moved farther than females—which may have increased their likelihood of encountering parasites (Brunner & Ostfeld, 2008). Male sex‐biased parasite diversity/load has been observed across a wide range of taxa (Moore & Wilson, 2002), including in white‐footed mice (Brunner & Ostfeld, 2008; Devevey & Brisson, 2012; Larson et al., 2018) and our results suggest that this phenomenon is likely caused by both direct and indirect effects. For example, direct effects of sex on tick occurrence may result from immunosuppressant effects of androgens or other hormones (Hughes & Randolph, 2001; Muehlenbein & Bribiescas, 2005) or occur because males forego investment in immunity and, instead, increase fitness by investing in higher mating rates (i.e., Bateman's principle; Rolff, 2002). When a tick attaches to a host for a blood meal, the host's immune system can mount a response against components found in the tick's saliva, which may result in premature drop‐off or even death of the tick in situ (Pollock et al., 2012) and such resistance can be acquired during prior tick infestations (Jones et al., 2015) (but see Ribeiro, 1989, as anti‐tick immunity in natural tick hosts such as *P. leucopus* may be weak). We observed higher rates of larval parasitism associated with reproductive state in males only; therefore, it is possible that, if elevated testosterone lowers the immune response of reproductive males, this has a greater affect than costly reproductive investment does in females (Christe et al., 2000; Moore & Wilson, 2002). Additionally, the lack of interaction between sex and reproduction on parasitism by nymphal ticks may indicate a less effective host immune response against nymphal ticks, or could arise because peak activity of the larval stage coincides with the timing of mouse reproduction (Hamer et al., 2012). Given the important differences in larval versus nymphal ticks in transmission of *B. burgdorferi* to humans (Tran et al., 2021), and evidence that nymphal ticks are more likely than larval ticks to be infected with tick‐borne pathogens that lack vertical transmission (Estrada‐Peña & De La Fuente, 2014), examining the relationship between immunity and stage‐specific parasitism may be a profitable avenue for future work. Moreover, as environmental variation can influence immune function, for example, via differences in diet composition (Blubaugh et al., 2023), seasonality (Versteegh et al., 2014), and local climate regimes (personal communication, Assis, et al., 2024), additional studies working at a broad geographic scope and across a range of environments and/or climates will be particularly important.

Indirect effects of sex on parasite attachment may arise due to sexually dimorphic space use, home range size, and differential dispersal in *P. leucopus* (Kirkland & Layne, 1989; Ostfeld, Miller, & Hazler, 1996; Wolff, 1985). We found that males moved farther between consecutive capture events than females and had higher trap diversity scores (i.e., were more likely to use a new trap upon subsequent captures than females). This observed increased movement by male *P. leucopus* likely leads to higher encounter rates with questing ticks, a possibility supported by our finding that greater movement distance is associated with higher individual prevalence of parasitism by both larval and nymphal ticks; Figure 3a,d, and also by the observation that this effect is stronger for nymphal than larval ticks. Since larval ticks are more clumped in space than nymphs (thousands having emerged from one single egg mass) (Devevey & Brisson, 2012), the effects of movement distance on tick encounters should be weaker for this life stage. Our results suggest that intraspecific variation in the movement ecology of hosts may yield unrecognized variation in host–parasite encounter rates.

Body mass was negatively related to parasitism by larval ticks, but positively related to parasitism by nymphal ticks (Figure 3b,e). This result may be a product of differing emergence times of larval and nymphal ticks (e.g., as in the blacklegged tick life cycle; Sandbergtii et al., 1992) and how this cycle coincides with the life cycle of *P. leucopus*. Although tick phenology may vary geographically (Ogden et al., 2018, 2021), larval ticks and subadult rodents (smaller than adults) are less abundant during the early spring when nymphs are actively seeking hosts, whereas many dispersing subadult mice are present during the mid to late summer period when larvae are most active (Lyman et al., 2001). Our finding that parasitism by nymphal ticks is higher in larger‐bodied individuals but parasitism by larval ticks is higher in smaller‐bodied individuals (Figure 3b,e) may reflect these phenological differences. Future work could further explore this potential role of phenological matching by examining ecoregions with differing rodent‐breeding phenologies (McLean & Guralnick, 2021).

Consistent differences in behaviors including movement activity, boldness, and sociality affect parasite transmission in a variety of host species (Hall, 2022). Although we did not find that trappability or trap diversity were related to parasitism in *P. leucopus*, several potential explanations exist that can help inform future efforts. First, high levels of variability in field‐observed rodent behavior (Brehm & Mortelliti, 2018) may make it difficult to resolve statistical relationships. Another possibility is that host space‐ and trap‐use behaviors may play an important role in an aspect of host–parasite interactions that we were unable to examine (e.g., tick burdens). Future work could examine whether trappability or trap diversity affect the total tick burden on an individual mouse, rather than the probability of being parasitized. Additionally, understanding how other ecologically relevant behaviors that we were unable to measure (i.e., grooming) may covary with metrics used in this study (such as age, sex, or movement behavior) may provide further insight.

### Local interactions also depend strongly on local conditions

Many zoonoses exist across large geographic scales (Han et al., 2016), spanning large‐scale variation in climate, habitat conditions, and host community assemblage. While our results indicate that host conditions and behavior may be useful for predicting large‐scale patterns in parasitism (Figure 2), they also indicate that local (i.e., site‐level) factors play a significant role in affecting variation in parasitism. Specifically, we used sampling grid as a random intercept in our models and this random‐effect term typically described a significant portion of the variance in the response variables in our SEM models (Figure 2). The importance of site‐level variation in factors other than host conditions and behavior suggests an important role for aspects of tick ecology in further honing models of host–parasite interactions. Ticks are highly aggregated in both space and time (Devevey & Brisson, 2012). Locally, aggregation of larvae may derive from several thousand larvae emerging from a single egg mass, and aggregation of nymphs is likely a consequence of where they fall from a single host after the larval blood meal (Devevey & Brisson, 2012). High spatial heterogeneity in tick abundance (locally and across the entire geographic range) may have limited our ability to identify strong predictive relationships between individual conditions, behavior, or forest context and the occurrence of parasitism. Given the potential for significant local variation in tick abundance and distribution, it is possible that individual conditions and behavior may be stronger predictors of parasitism by ticks, but only in locations where ticks are present in high densities. Sampling of ticks is not performed on the same grids where NEON samples small mammals, limiting our ability to test this possibility. Working at two sites within the same locality where they also quantified the spatial aggregation of host‐seeking *I. scapularis* larvae and nymphs, Devevey and Brisson (2012) concluded that host characteristics, not tick aggregation, played an essential role in variation in tick burdens among hosts. This suggests that future studies that link rodent behavior and conditions (Figure 2) with local data on tick abundance and distribution could help provide even greater predictive power regarding host–parasite dynamics and disease prevalence.

### Implications for zoonotic disease systems

Our finding that the magnitude of individual host movements affects the occurrence of parasitism suggests that factors influencing space use (such as resource availability, habitat fragmentation, or predator reintroductions) may play a role in zoonotic disease transmission rates (Buchmann et al., 2013; Preisser et al., 2007; Smith et al., 2019; Stradiotto et al., 2009; Svoboda et al., 2019; Wolff, 1985). For example, invasion by exotic shrubs such as *Rhamnus cathartica*, or buckthorn, are shown to alter the movement ecology of *P. leucopus*, leading to increased space‐use and disrupting the use of coarse woody debris as refuge (Guiden & Orrock, 2017). If mice in invaded forests move farther, our results suggest that these mice will also have increased prevalence of parasitism by *Ixodid* ticks and perhaps tick‐borne pathogens.

Tick‐borne diseases pose significant threats to human health worldwide and annual reports of tick‐borne diseases have more than doubled since 2004 (Rosenberg et al., 2018). Lyme disease accounted for more than 80% of all tickborne disease reports in the US during 2004–2016, and increasing prevalence of this disease in historically Lyme‐free regions such as the Great Lakes region and southern Quebec has been attributed to northward expansion by *P. leucopus* (Roy‐Dufresne et al., 2013). Further, *Ixodid* ticks and the diseases they transmit are inherently sensitive to climate, which is thought to contribute to observed poleward range expansion and increased abundance of ticks in many regions (Ogden et al., 2021). It is increasingly important to understand the ecological and behavioral factors that drive encounters between vectors and hosts, particularly as *P. leucopus* is just one member of a complex community of tick hosts and other zoonotic reservoirs (Brunner et al., 2008). For example, the woodland deer mouse, *P. maniculatus*, may serve as another important reservoir of tick‐borne pathogens (Larson et al., 2018, 2021). This species is widely captured by NEON across North America. Although we were unable to examine this species due to limited sample sizes (see Appendix S6: Table S1, Figure S1), ongoing NEON sampling continues to generate data that should help ameliorate sample‐size limitations in the future. Moreover, additions like increased molecular testing (for positive species identification) and plans to quantify tick burdens will also produce data that empower additional analyses. Particularly, comparisons of reservoirs with varying space use and habitat associations, such as *P. maniculatus*, which are more arboreal than *P. leucopus* (Kirkland & Layne, 1989), will allow us to better understand associations between animal movements and zoonotic disease risk, as arboreal movements may limit encounters with ground‐questing *Ixodid* ticks.

### Concluding remarks and future directions

The factors that shape encounters between disease vectors and wildlife hosts affect the transmission of zoonotic pathogens (Devevey & Brisson, 2012) and the success of management efforts (Tsao et al., 2021). Understanding how local parasite aggregation and density affect the strength of associations between host behavior and parasite encounter rates remains an important step for future research, and including other host species with varying movement ecologies should continue to elucidate the role that individual host heterogeneity plays in zoonotic diseases. In demonstrating widespread associations between behavior and parasitism, our work suggests that studies seeking to link behavior to other aspects of zoonotic disease across broad geographic scales (e.g., infection) could be very informative. As humans continue to modify landscapes in ways that alter the movement ecology and space‐use of individuals, such as by introducing invasive plants (Guiden & Orrock, 2017), or changing predator communities (Wirsing et al., 2021) our findings suggest that these modifications may have unappreciated consequences on parasite acquisition and, ultimately, pathogen transmission. Furthermore, the magnitude of host movements is only important for host–vector encounter rates if hosts are active at the same time as vectors. Invasive shrubs, for example, are shown not only to alter space‐use, but the timing of rodent activity (Guiden & Orrock, 2019); allowing rodents to become active earlier and stay active longer by dampening moon illumination. Future work should incorporate rodent activity timing among varied landscapes in models predicting parasitism by ticks and infection rates.

Adding to a breadth of research performed at local scales, our work has uncovered that generally supported trends such as sex‐biased parasitism, increased prevalence of parasites in reproductive males, and higher host‐tick encounter rates with greater ranging behavior are apparent across much of the US. Furthermore, we tease apart the direct and indirect effects of such factors in driving parasite acquisition by mice, identifying that male‐biased parasitism reflects drivers that are separate from sexually dimorphic morphology (i.e., body size) or behavior (space‐use).

## AUTHOR CONTRIBUTIONS

Allison M. Brehm and John L. Orrock conceived the study, Allison M. Brehm and John L. Orrock conceived the study methodology with input from Lynn B. Martin and Vania R. Assis, Allison M. Brehm performed data extraction, processing, and analysis, Allison M. Brehm drafted the initial manuscript with input from John L. Orrock, all authors contributed critically to manuscript revision and final draft preparation.

## CONFLICT OF INTEREST STATEMENT

The authors declare no conflicts of interest.

## Supporting information


Appendix S1.

Appendix S2.

Appendix S3.

Appendix S4.

Appendix S5.

Appendix S6.

## Data Availability

The following National Ecological Observatory Network (NEON) datasets were utilized for this research: small mammal box trapping (National Ecological Observatory Network, 2023b), https://doi.org/10.48443/p4re-p954; ticks sampled using drag cloths (National Ecological Observatory Network, 2023c), https://doi.org/10.48443/3zmh-xx57; NEON Biorepository Mammal Collection (DNA Extracts) occurrence dataset (National Ecological Observatory Network, 2023a), https://doi.org/10.15468/6mxmvr.
