# Supplementary material for: Individual variation underlies large‐scale patterns: Host conditions and behavior affect parasitism
Source: Ecology. 2024 Dec 9;106(1):e4478. doi: 10.1002/ecy.4478 (PMC11739666; doi:10.1002/ecy.4478)
Supplement: Supplementary file 1 — Appendix S1. [file ECY-106-e4478-s002.pdf]

Journal: Ecology

Title: Individual variation underlies large-scale patterns: Host conditions and behavior affect parasitism

Authors: Allison M. Brehm, Vania R. Assis, Lynn B. Martin, and John L. Orrock

Appendix S1 – A three-step process to confirm taxonomic identification of *Peromyscus leucopus* used in SEM analyses

Although NEON stores tissue samples from each captured individual at the Arizona State University Biorepository (https://biorepo.neonscience.org/portal/), only a subset of samples undergo DNA extraction for genetic testing and receive a confirmed taxonomic identification. Therefore, step one was to confirm species identification for all individuals whose tissue samples underwent genetic testing at the biorepository (NEON DNA data barcoding data product DP1.10076.001). Individuals that were genetically confirmed as *P. leucopus* (with ≥98% sequence match between the barcode and the top GenBank nucleotide number from a NCBI BLAST search) were retained in the dataset (1132 individuals). Step two was to compare taxonomic identification performed in the field with the genetic taxonomic identification from the biorepository. This allowed us to assess which NEON sites have high classification accuracy in the field (i.e., never misidentified a sample), and which sites only ever captured one species of *Peromyscus* (Figures S1-S9). We updated taxonomic identification in the mammal trapping data accordingly and retained individuals reclassified as *P. leucopus* in the dataset (6241 individuals). In step three, we used morphometric measures of individuals with genetic taxonomic ID from the biorepository to develop discriminate function analysis (DFA) equations to identify characteristics that best distinguished *P. leucopus* from *P. maniculatus* (Stephens et al., 2014) and classified individuals with uncertain taxonomy using these equations (1026 individuals). For a detailed description of this process and the DFA equations, see Boxes S1 and S2.

Step 1**:** The NEON DNA data barcoding data product (DP1.10076.001, accessed 6/15/2023) contains 1424 records of barcoded individuals with confirmed taxonomic identification of *P. leucopus* (with high confidence; i.e., a ≥98% sequence match between the barcode and the top GenBank nucleotide number from a NCBI BLAST search). After data filtering described in the methods, 1132 of these individuals were in the dataset, and this confirmed species identification was added. We reclassified these individuals as PELE even if they were field-classified as another species (See examples in step 2 below).

Step 2**:** We compared taxonomic identification performed in the field with the genetic taxonomic identification from the biorepository (again, using only barcoded specimens with ≥98% sequence match) to assess which NEON sites have high classification accuracy in the field (i.e., never misidentified a sample or only misidentified a small fraction of individuals), and which sites only ever captured one species of *Peromyscus*. We updated taxonomic identification in the mammal trapping data accordingly and retained individuals reclassified as *P. leucopus* in the dataset. Out of 16 total NEON sites used in this study, we were able to make the following 9 assumptions from 9 sites with high classification accuracy and/or only one species of *Peromyscus* present:

1: All barcoded *Peromyscus* specimens at the Blandy Experimental Farm NEON site (BLAN) were taxonomically identified as PELE (*Peromyscus leucopus*). Any individual field-classified as PEMA (*Peromyscus maniculatus*) or PESP (*Peromyscus spp*.) at BLAN is very likely a PELE, and the data can be corrected accordingly (Figure S1).

Figure S1. Genetic taxonomic classification of all *Peromyscus* with barcode data. At Blandy Experimental Farm (BLAN), NEON has barcode data for 81 individuals. All 81 individuals were genetically identified as *Peromyscus leucopus (*PELE*)*. Of these individuals, 75 had been classified in the field as PELE, 5 had been classified at *Peromyscus maniculatus* (PEMA), and 1 had been classified as PESP (*Peromyscus* spp.).

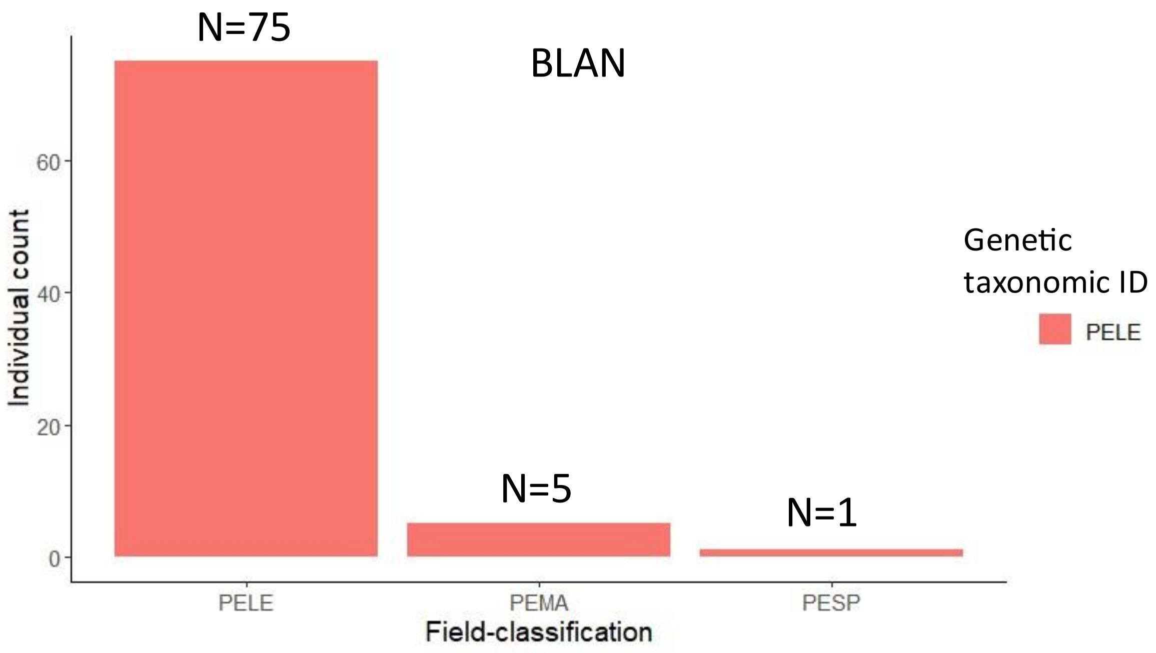

2: All barcoded *Peromyscus* specimens at the Harvard Forest NEON site (HARV) were taxonomically identified as PELE. Any individual field-classified as PEMA, PESP, or PELEPEMA (either *P. leucopus* or *P. maniculatus*) at HARV is very likely a PELE, and the data can be corrected accordingly (Figure S2).

Figure S2. Genetic taxonomic classification of all *Peromyscus* with barcode data. At Harvard Forest (HARV), NEON has barcode data for 177 individuals. All 177 individuals were genetically identified as *Peromyscus leucopus (*PELE*).* Of these individuals, 73 had been classified in the field as PELE, 67 had been classified as *Peromyscus maniculatus* (PEMA), 34 had been classified as either PELE or PEMA (PELEPEMA) and 3 had been classified as PESP (*Peromyscus* spp.).

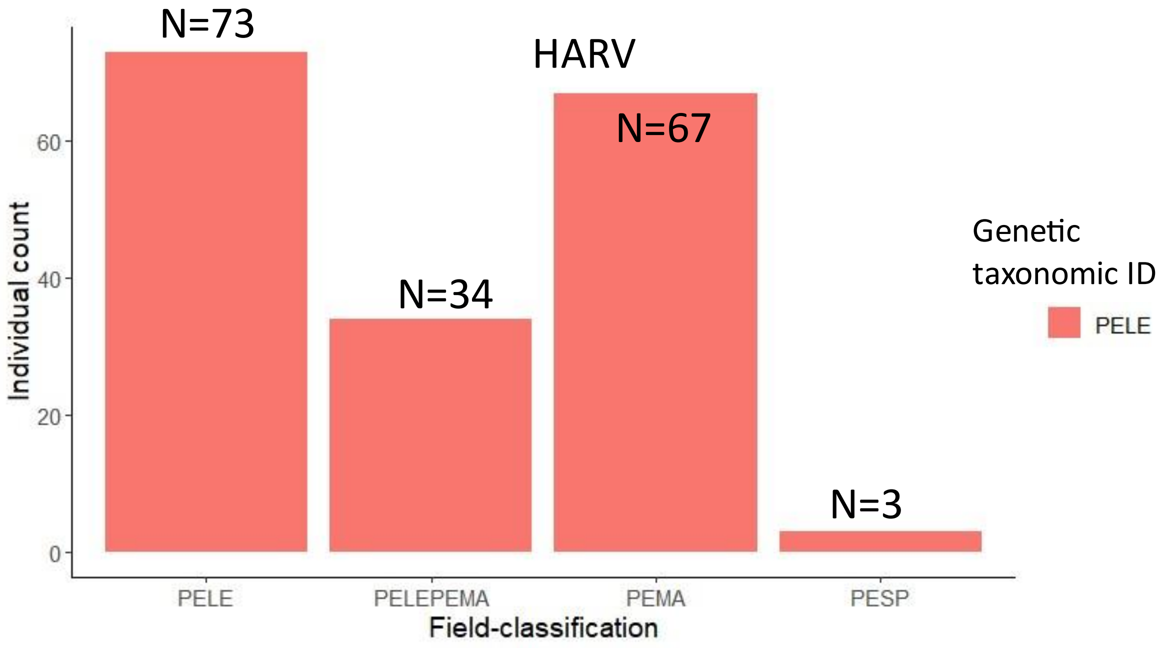

3: All barcoded *Peromyscus* specimens at the Smithsonian Conservation Biology Institute NEON site (SCBI) were taxonomically identified as PELE. Any individual field-classified as PEMA at SCBI is very likely a PELE, and the data can be corrected accordingly (Figure S3).

Figure S3. Genetic taxonomic classification of all *Peromyscus* with barcode data. At Smithsonian Conservation Biology Institute (SCBI), NEON has barcode data for 145 individuals. All 145 individuals were genetically identified as *Peromyscus leucopus (*PELE*).* Of these individuals, 127 had been classified in the field as PELE, and 18 had been classified as *Peromyscus maniculatus* (PEMA).

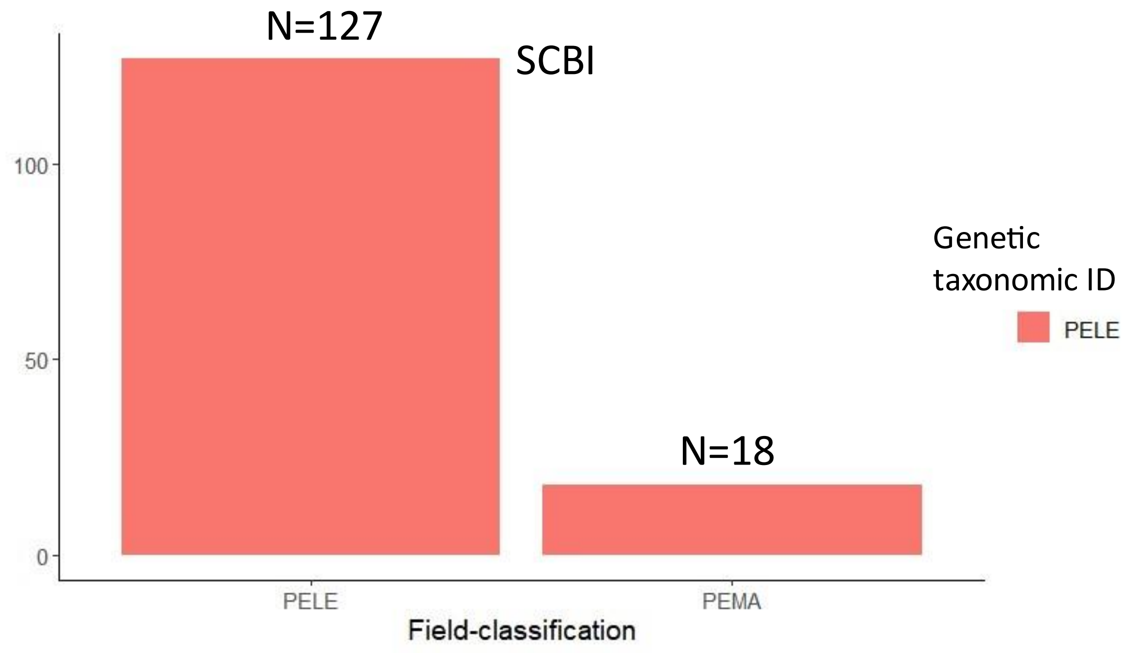

4: All barcoded *Peromyscus* specimens at the Smithsonian Environmental Research Center NEON site (SERC) were taxonomically identified as PELE. Any individual field-classified as PEMA at SERC is very likely a PELE, and the data can be corrected accordingly (Figure S4).

Figure S4. Genetic taxonomic classification of all *Peromyscus* with barcode data. At Smithsonian Environmental Research Center (SERC), NEON has barcode data for 91 individuals. All 91 individuals were genetically identified as *Peromyscus leucopus (*PELE*).* Of these individuals, 85 had been classified in the field as PELE, 5 had been classified as *Peromyscus maniculatus* (PEMA), and 1 had been classified as *Peromyscus spp. (*PESP).

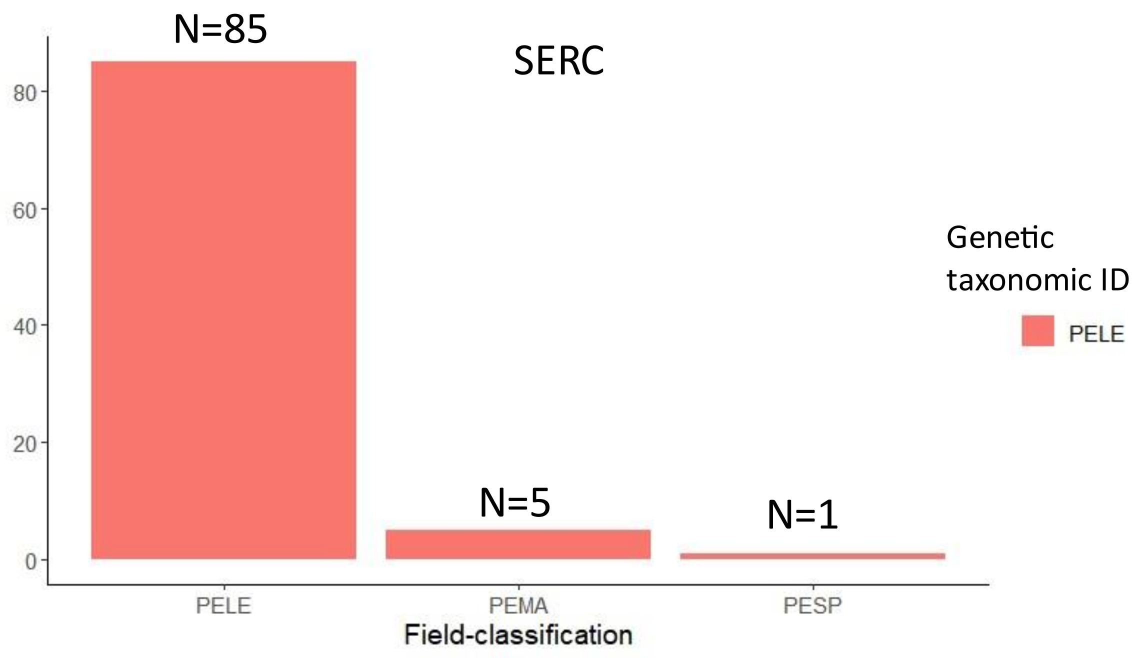

5: There was high accuracy of field classification for PELE at the Great Smoky Mountains National Park NEON site (GRSM). Any individual field-classified as PELE at GRSM is likely to be correctly classified (Figure S5). Individuals field-classified as PEMA but confirmed genetically to be PELE (with ≥98% sequence match) were retained in the dataset.

Figure S5. Genetic taxonomic classification of all *Peromyscus* with barcode data. At Great Smoky Mountains National Park (GRSM), NEON has barcode data for 117 individuals. Of 69 individuals classified in the field as *Peromyscus leucopus (*PELE*)*, 64 of these individuals were genetically confirmed as PELE, 2 as *Peromyscus maniculatus* (PEMA), and 3 as *Peromyscus gossypinus*. *(*PEGO). Of 48 individuals classified in the field as PEMA, 23 were classified genetically (with ≥98% sequence match) as PELE.

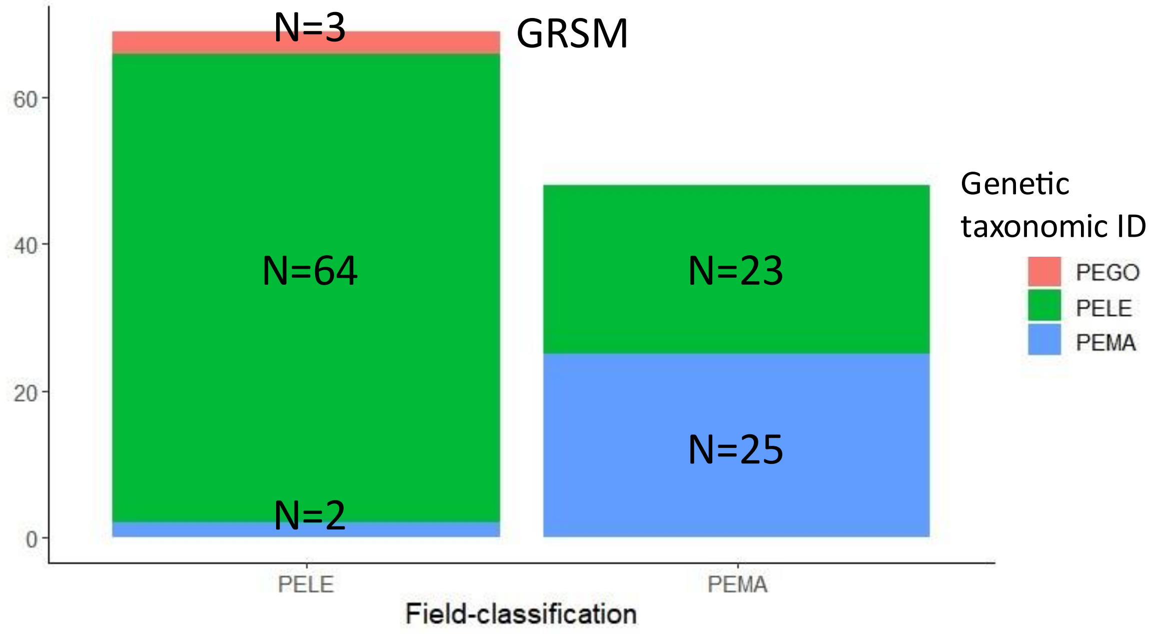

6: There was high accuracy of field classification for PELE at the Konza Prairie Biological Station NEON site (KONZ). Any individual field-classified as PELE at KONZ is likely to be correctly classified (Figure S6). Individuals field-classified as PEMA but confirmed genetically to be PELE (with ≥98% sequence match) were retained in the dataset.

Figure S6. Genetic taxonomic classification of all *Peromyscus* with barcode data. At Konza Prairie Biological Station (KONZ), NEON has barcode data for 110 individuals. Of 59 individuals classified in the field as *Peromyscus leucopus (*PELE*)*, 56 of these individuals were genetically confirmed as PELE, and 3 as *Peromyscus maniculatus* (PEMA). Of 51 individuals classified in the field as PEMA, 12 were classified genetically (with ≥98% sequence match) as PELE.

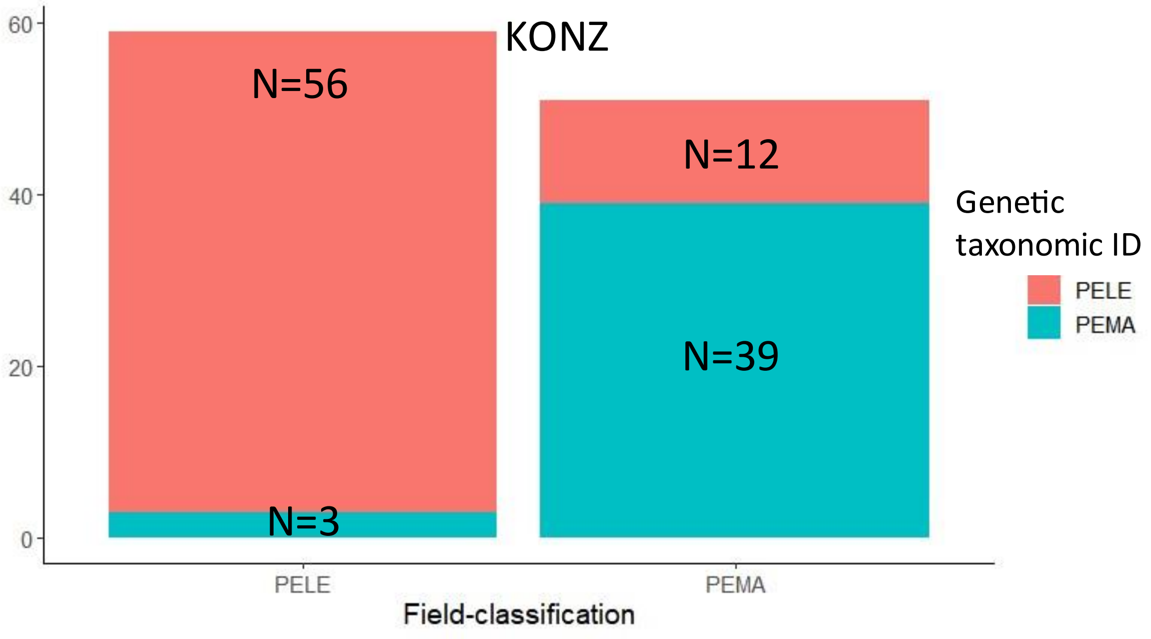

7: There was high accuracy of field classification for PELE at the Mountain Lake Biological Station NEON site (MLBS). Any individual field-classified as PELE at MLBS is likely to be correctly classified (Figure S7). Individuals field-classified as PEMA but confirmed genetically to be PELE (with ≥98% sequence match) were retained in the dataset.

Figure S7. Genetic taxonomic classification of all *Peromyscus* with barcode data. At Mountain Lake Biological Station (MLBS), NEON has barcode data for 128 individuals. Of 70 individuals classified in the field as *Peromyscus leucopus (*PELE*)*, 67 of these individuals were genetically confirmed as PELE, and 3 as *Peromyscus maniculatus* (PEMA). Of 58 individuals classified in the field as PEMA, 31 were classified genetically (with ≥98% sequence match) as PELE.

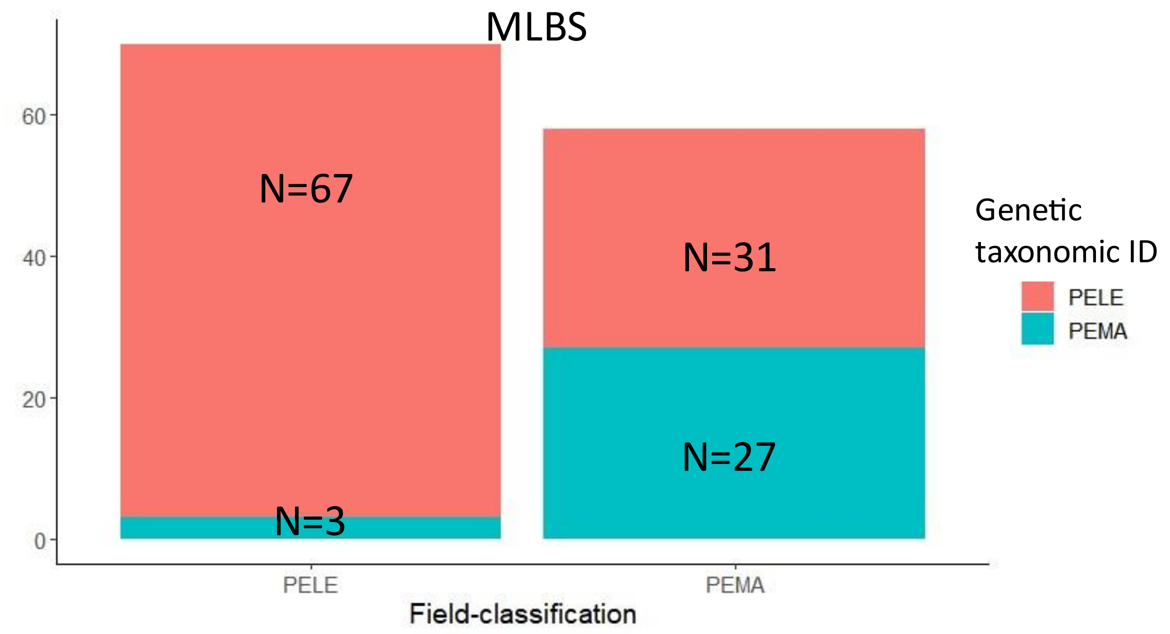

8: There was high accuracy of field classification for PELE at the Oak Ridge National Laboratory NEON site (ORNL). Any individual field-classified as PELE at ORNL is likely to be correctly classified (Figure S8). Individuals field-classified as PEMA but confirmed genetically to be PELE (with ≥98% sequence match) were also retained in the dataset.

Figure S8. Genetic taxonomic classification of all *Peromyscus* with barcode data. At Oak Ridge National Laboratory (ORNL), NEON has barcode data for 102 individuals. Of 68 individuals classified in the field as *Peromyscus leucopus (*PELE*)*, 66 of these individuals were genetically confirmed as PELE, and 2 as *Peromyscus gossypinus* (PEGO). Of 34 individuals classified in the field as *Peromyscus maniculatus* (PEMA), 33 were classified genetically (with ≥98% sequence match) as PELE, and 1 as PEGO.

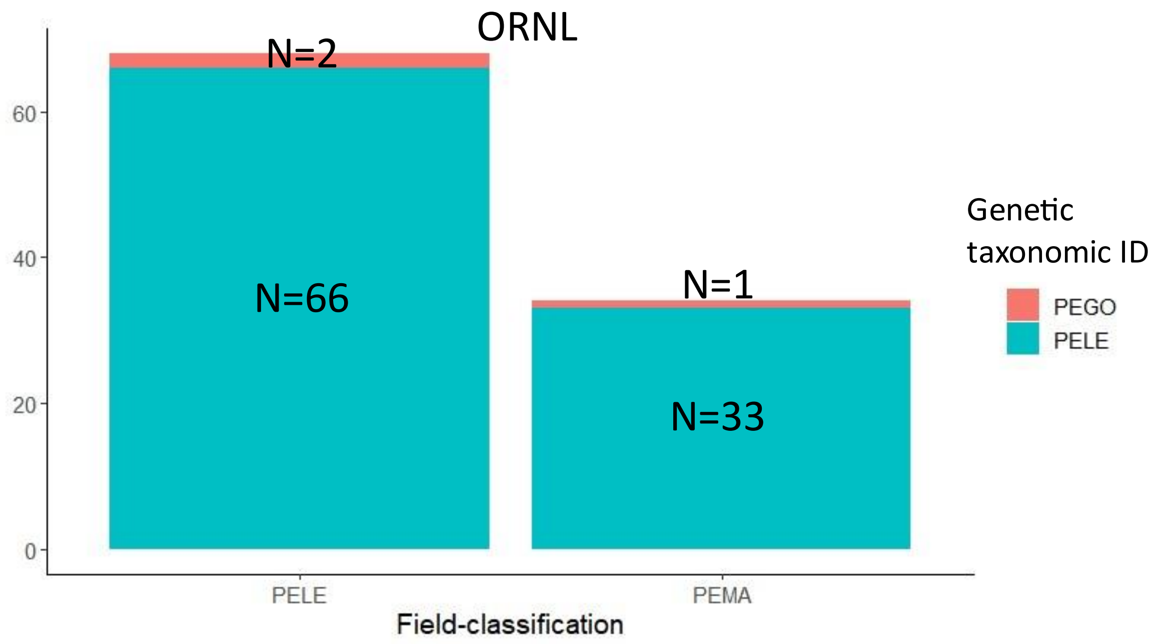

9: There was high accuracy of field classification for PELE at the University of Kansas Field Station NEON site (UKFS). Any individual field-classified as PELE at UKFS is likely to be correctly classified (Figure S9). Individuals field-classified as PEMA but confirmed genetically to be PELE (with ≥98% sequence match) were also retained in the dataset.

Figure S9. Genetic taxonomic classification of all *Peromyscus* with barcode data. At the University of Kansas Field Station (UKFS), NEON has barcode data for 64 individuals. Of 52 individuals classified in the field as *Peromyscus leucopus (*PELE*)*, all of these individuals were genetically confirmed as PELE. Of 12 individuals classified in the field as *Peromyscus maniculatus* (PEMA), 2 were classified genetically (with ≥98% sequence match) as PELE.

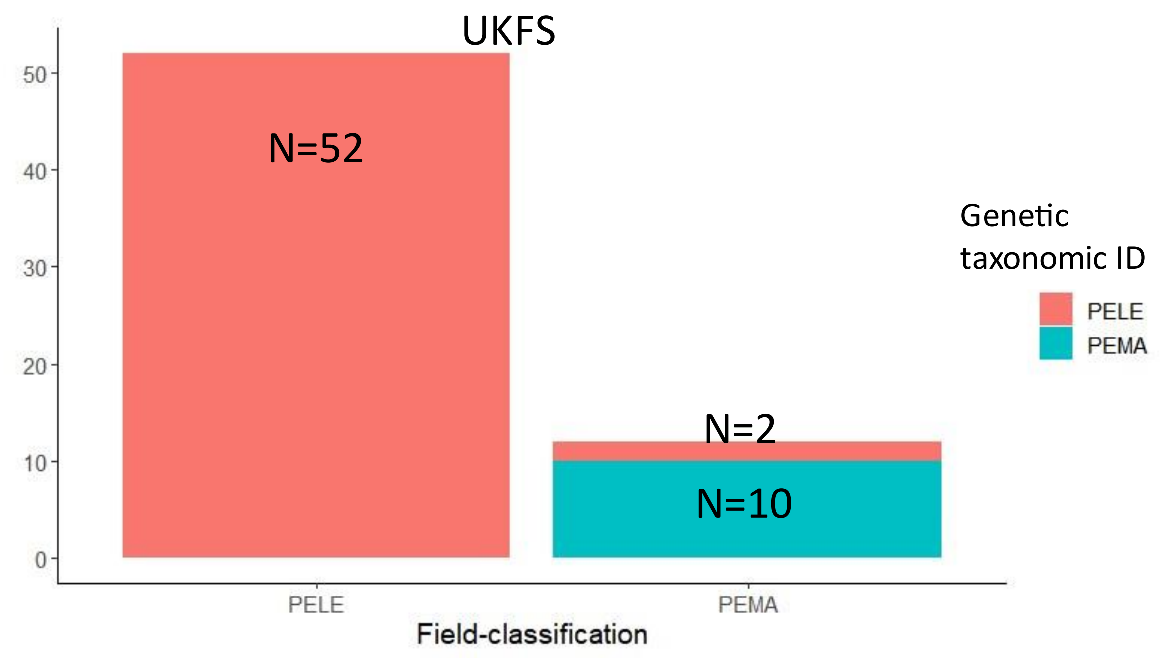

Step 3: We used the morphometric measures from individuals with genetic taxonomic ID (with ≥98% sequence match) from the biorepository to develop discriminate function analysis (DFA) equations using the "MASS" package in program R (Ripley et al. 2013) to identify characteristics that best distinguished *P. leucopus* from *P. maniculatus* and classified individuals with uncertain taxonomy using these equations. Individuals who were classified as *P. leucopus* using these equations were added to the final SEM dataset (1026 individuals total). The morphometric measures collected by NEON (description of procedures and protocols can be found in the NEON Doc. # NEON.DOC.000481) are as follows: ear length (length from notch at base of ear to end of pinna), hind foot length (length from the back of the heel to the end of the longest claw beyond the fleshy toe), tail length (length from bend on the back at the base of the tail to the tip of the fleshy part of the tail when tail is held at 90 degree angle to body, excluding projecting hairs), total length (length from tip of nose to tip of the fleshy part of the tail when animal's body is pressed flat and tail is straight and taught), body length (total length – tail length), body mass (mass in grams), tail/body ratio (ratio of tail length to body length).

We developed two separate DFA equations:

Equation 1: Species ~ ear length + tail length + body mass + tail/body ratio

There were 139 barcoded specimens with all four of the above morphometric measurements taxonomically identified as either PELE or PEMA. We randomly selected 75% of these mice (the training data), estimated the above parameters on this training data, and then used discriminant function to classify the remaining 25% of individuals (the holdout sample). We repeated this process 100 times, randomly selecting a different 75% of individuals on each iteration.

Box S1. Code for running linear discriminant analysis (Equation 1) to classify individuals with uncertain taxonomy. Line numbers added for reference. Analysis performed using the "MASS" package in program R (Ripley et al. 2013).

```
n=139 # total number of individuals with values for morphometric measures used in Equation 1
nt=104 # 75% of n to be used as the training data
neval=n-nt
rep=100 # number of repetitions to be performed
library(MASS) # load MASS package to perform linear discriminant analysis (LDA)
### LDA
set.seed(123456789)
errlin=dim(rep)
for (k in 1:rep) {
  train=sample(1:n,nt)
  ## linear discriminant analysis
  m1 = lda(DNAsh ~ earLength + tailLength + weight + tail_length_ratio, data=pemapele_nonas_4_first[train,])
  predict(m1, pemapele_nonas_4_first[-train,])$class
  tablin=table(pemapele_nonas_4_first$DNAsh[-train],
  predict(m1,pemapele_nonas_4_first[-train,])$class)
  errlin[k]=(neval-sum(diag(tablin)))/neval
}
merrlin=mean(errlin) # calculate mean of the error rate across 100 runs
merrlin
rerrlin=range(errlin) # calculate the range of the error rate across 100 runs
rerrlin
```

In the above example, merrlin = 0.1168. This means that there was, on average, an 11.7% misclassification rate on the holdout data, and 88.3% of individuals were classified correctly. The range for this error rate (rerrlin) equals 0-0.2. We then trained this discriminant function equation using all 139 genetically classified individuals and predicted species classification for the individuals in the SEM dataset with values for all four morphometric measures (230 individuals). Of these 230 individuals, 103 were assigned a confirmed species classification in

either *Step 1* or *Step 2*, and the discriminant function equation correctly classified these individuals 97% of the time, confirming high accuracy of the equation.
Equation 2: Species ~ tail length

There were 1172 barcoded specimens with a measure for tail length taxonomically identified as either PELE or PEMA. We randomly selected 75% of these mice (the training data), estimated the above parameters on this training data, and then used discriminant function to classify the remaining 25% of individuals (the holdout sample). We repeated this process 100 times, randomly selecting a different 75% of individuals on each iteration.

Box S2. Code for running linear discriminant analysis (Equation 2) to classify individuals with uncertain taxonomy. Line numbers added for reference. Analysis performed using the "MASS" package in program R (Ripley et al. 2013).

```
n=1172 # total number of individuals with values for morphometric measures used in Equation 1
nt=879 # 75% of n to be used as the training data
neval=n-nt
rep=100 # number of repetitions to be performed
library(MASS) # load MASS package to perform linear discriminant analysis (LDA)
### LDA
set.seed(123456789)
errlin=dim(rep)
for (k in 1:rep) {
  train=sample(1:n,nt)
  ## linear discriminant analysis
  m1 = lda(DNAsh ~ tailLength, data=pemapele_nonas_1_first[train,])
  predict(m1, pemapele_nonas_1_first[-train,])$class
  tablin=table(pemapele_nonas_1_first$DNAsh[-train],
  predict(m1,pemapele_nonas_1_first[-train,])$class)
  errlin[k]=(neval-sum(diag(tablin)))/neval
}
merrlin=mean(errlin) # calculate mean of the error rate across 100 runs
merrlin
rerrlin=range(errlin) # calculate the range of the error rate across 100 runs
rerrlin
```

In the above example, merrlin = 0.279. This means that there was a 27.9% misclassification rate on the holdout data, and 72.1% of individuals were classified correctly. The range for this error rate (rerrlin) equals 0.225-0.348 We then trained this discriminant function equation using all 1172 genetically classified individuals and predicted species classification for the individuals in the SEM dataset with values for tail length (4638 individuals). Of these 4638 individuals, 3704 were assigned a confirmed species classification in either *Step 1*, *Step 2*, or part 1 of *Step 3* and the discriminant function equation correctly classified these individuals 95% of the time, confirming high accuracy of the equation.

Summary of sample after each step:
Step 1 – 1132 confirmed PELE
Step 2 – 7373 confirmed PELE
Step 3 – *Equation 1* - 7491 confirmed PELE
Equation 2 - 8399 confirmed PELE
*Note, the above sample of 8399 individuals still contains individuals with missing values for distance moved, etc. Further data filtering is detailed in the main text, methods subsection “Structural equation models (SEM)”. Specifically, we state that “we extracted all complete observations for the following variables (sex, average body mass, average movement distance, proportion of captures reproductive, parasitism by larval ticks, parasitism by nymphal ticks, trappability, and trap diversity).”

References

Ripley, B., Venables, B., Bates, D. M., Hornik, K., Gebhardt, A., Firth, D., & Ripley, M. B. (2013). Package ‘mass’. Cran R, 538, 113–120.

Stephens, R. B., Anderson, E. M., Wendt, S. R., & Meece, J. K. (2014). Field identification of sympatric *Peromyscus leucopus noveboracensis* and *P. maniculatus gracilis* in Wisconsin from external measurements. *The American Midland Naturalist*, *171*(1), 139–146. https://doi.org/10.1674/0003-0031-171.1.139
