# Supplementary material for: Individual variation underlies large‐scale patterns: Host conditions and behavior affect parasitism
Source: Ecology. 2024 Dec 9;106(1):e4478. doi: 10.1002/ecy.4478 (PMC11739666; doi:10.1002/ecy.4478)

Journal: Ecology

Title: Individual variation underlies large-scale patterns: Host conditions and behavior affect parasitism

Authors: Allison M. Brehm, Vania R. Assis, Lynn B. Martin, and John L. Orrock

Appendix S2

To assess whether movement distance measures should be limited to within-session movements only, we estimated movement across all consecutive captures (both within and across sessions), specifying “within” vs “across” as a grouping variable called “measure type”. We then partitioned the variance with a linear mixed effects model with distance as the response variable, and both individual identity and measure type as random intercepts. The proportion of variance in distance that was attributed to measure type was less than 0.01%, suggesting that it is not necessary to consider statistically whether a measure was taken from within vs across session capture events.

Table S1. Results of variance partitioning using measure type (within vs across session movements) and individual identity as grouping variables.

Linear mixed model fit by REML [‘lmerMod’]
Formula: dist ~ 1 + (1|id) + (1|measure)

Random effects:		
Group	*Variance*	*Std. Dev*
Id	1446	38.03
Measure	0.00002	1.28
Residual	3025	55.00

Number of obs: 10130, groups: id, 4102; measure, 2

Repeatability of measure = 0.00002 / (0.00002 + 1446 + 3025) = 4.47e-9
Less than 0.01 % of the variance in "dst" is attributable to differences between within and among-session measurements
Repeatability of id = 1446/ (0.00002 + 1446 + 3025) = 0.32 (32% due to differences between individuals)

Figure S1. Distribution of movements made by individual white-footed mice, *Peromyscus leucopus*. Movement distance (dist) represents the distance between consecutive capture locations (in meters). Movements made within a single trapping session (within) vs across trapping sessions (across) are similarly distributed.

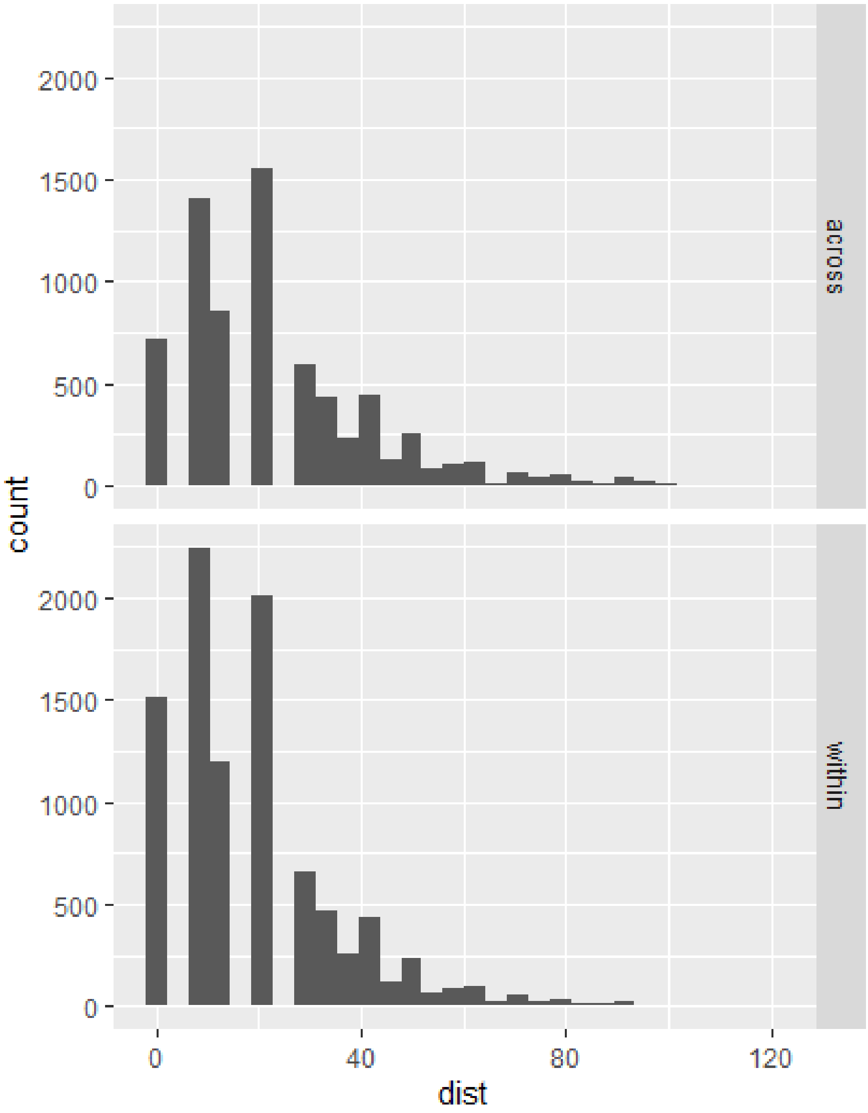

Supplement: Supplementary file 2 — Appendix S2. [file ECY-106-e4478-s006.pdf]
