# Supplementary material for: Individual variation underlies large‐scale patterns: Host conditions and behavior affect parasitism
Source: Ecology. 2024 Dec 9;106(1):e4478. doi: 10.1002/ecy.4478 (PMC11739666; doi:10.1002/ecy.4478)

Journal: Ecology

Title: Individual variation underlies large-scale patterns: Host conditions and behavior affect parasitism

Authors: Allison M. Brehm, Vania R. Assis, Lynn B. Martin, and John L. Orrock

Appendix S3

Figure S1. Structural equation model (SEM) meta model structure for identifying direct and indirect relationships between morphology, behavior, parasitism by ticks, and forest context in the white-footed mouse, *Peromyscus leucopus.*

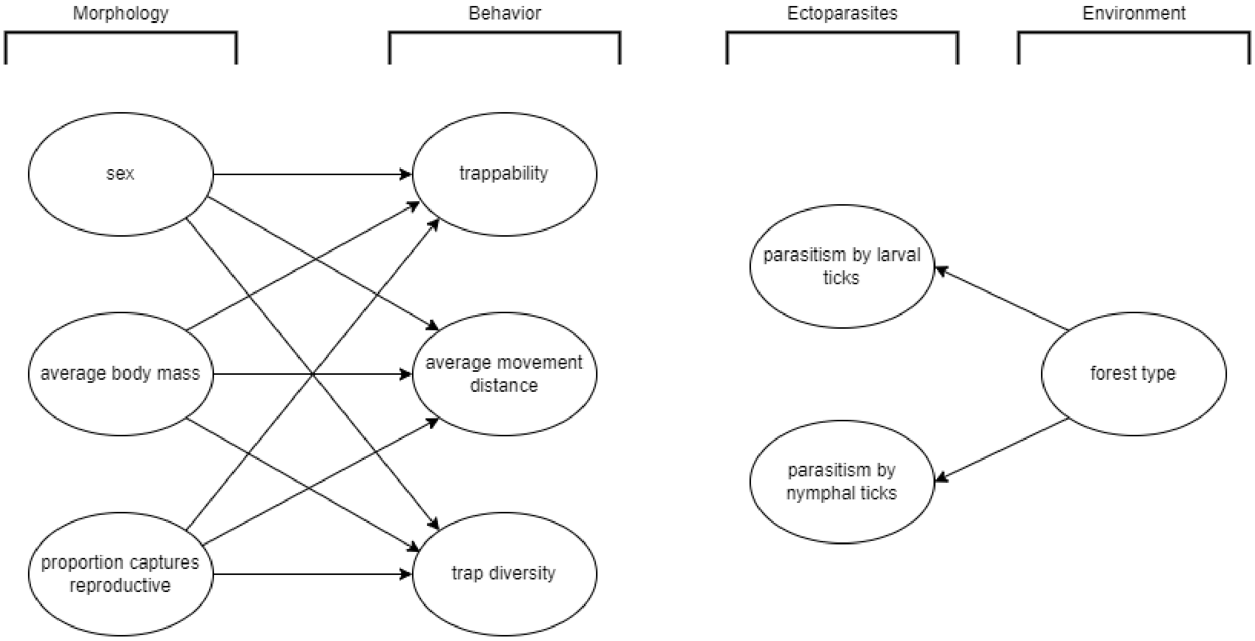

Supplement: Supplementary file 3 — Appendix S3. [file ECY-106-e4478-s004.pdf]
