# Supplementary material for: Individual variation underlies large‐scale patterns: Host conditions and behavior affect parasitism
Source: Ecology. 2024 Dec 9;106(1):e4478. doi: 10.1002/ecy.4478 (PMC11739666; doi:10.1002/ecy.4478)
Supplement: Supplementary file 4 — Appendix S4. [file ECY-106-e4478-s005.pdf]

Journal: Ecology

Title: Individual variation underlies large-scale patterns: Host conditions and behavior affect parasitism

Authors: Allison M. Brehm, Vania R. Assis, Lynn B. Martin, and John L. Orrock

Appendix S4

Table S1. Summary of variables used in piecewise structural equation models for white footed mice (*P. leucopus*)

Variable	Mean	Range	SD
Tick parasitism[1]	0.36	(0, 1)	0.38
Parasitism by larval ticks[2]	0.31	(0, 1)	0.37
Parasitism by nymphal ticks[3]	0.09	(0, 1)	0.20
Proportion captures reproductive[4]	0.45	(0, 1)	0.40
Trappability	0.73	(0.06, 1)	0.22
Trap diversity	0.87	(0.25, 1)	0.18
Mean body mass	21.35	(10.5, 38.5)	4.24
Mean distance moved	21.37	(0, 120.41)	14.59

[1]Proportion of captures individual was observed with ≥ one tick attached
[2]Proportion of captures individual was observed with ≥ one larval tick attached
[3]Proportion of captures individual was observed with ≥ one nymphal tick attached
[4]Proportion of captures individual was observed in a reproductive state
