# Supplementary material for: Individual variation underlies large‐scale patterns: Host conditions and behavior affect parasitism
Source: Ecology. 2024 Dec 9;106(1):e4478. doi: 10.1002/ecy.4478 (PMC11739666; doi:10.1002/ecy.4478)

Journal: Ecology

Title: Individual variation underlies large-scale patterns: Host conditions and behavior affect parasitism

Authors: Allison M. Brehm, Vania R. Assis, Lynn B. Martin, and John L. Orrock

Appendix S5

Figure S1. Predicted relationships (and 95% CI) between individual state and behavior in the white footed mouse, *Peromyscus leucopus*. (a-c) Male mice move farther on average than females and have greater trappability and trap diversity. (a) Body mass is positively related to the average distance moved between consecutive captures, but (b,c) negatively related to trappability and trap diversity. Trappability and trap diversity have been back-transformed from a logit-transformation.

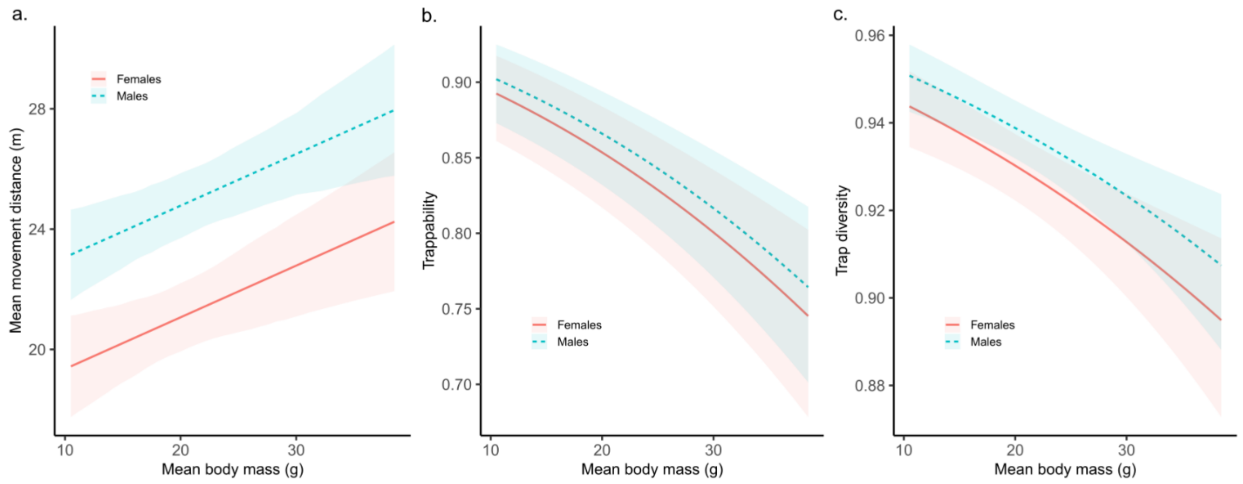

Supplement: Supplementary file 5 — Appendix S5. [file ECY-106-e4478-s003.pdf]
