# Supplementary material for: Individual variation underlies large‐scale patterns: Host conditions and behavior affect parasitism
Source: Ecology. 2024 Dec 9;106(1):e4478. doi: 10.1002/ecy.4478 (PMC11739666; doi:10.1002/ecy.4478)

Journal: Ecology

Title: Individual variation underlies large-scale patterns: Host conditions and behavior affect parasitism

Authors: Allison M. Brehm, Vania R. Assis, Lynn B. Martin, and John L. Orrock

Appendix S6

Table S1. Generalized linear mixed effects model structure predicting parasitism by ticks in the woodland deer mouse, *Peromyscus maniculatus*. Model specified with family = "binomial". Small sample sizes and low rates of tick attachment may limit our ability to find relationships between host state, behavior, forest type, and parasitism by ticks. Number of individuals = 192, number of trapping grids = 35. All continuous predictor variables are scaled and centered.

**Random effects**			
Group	Variance	Std.Dev.	
Trapping grid	0.06047	0.2459	
**Fixed effects**			
Variable	Estimate	St. Error	*p*-value
(Intercept)	-1.3051	0.3487	0.000182
[1]Avg. movement distance	-0.2411	0.1936	0.21288
Sex	0.6232	0.3686	0.090905
Avg. body mass	0.1555	0.2181	0.475728
[2]Deciduous	-0.3617	0.3808	0.342239
[3]Proportion captures reproductive	-0.2805	0.2293	0.221241

[1]Average distance moved between consecutive captures (in meters)

[2]Binary variable indicating whether the trapping grid was located in deciduous forest or not

[3]The proportion of captures during which an individual was observed in a reproductive state

The response variable is a binary (0,1) indicating whether an individual was ever observed with a tick of any life stage attached.

Figure S1. The majority of individual deer mice, *Peromyscus maniculatus*, trapped by NEON (and that met the data filtering requirements outlined in the main text) were not ever observed with a tick (of any life stage) attached.

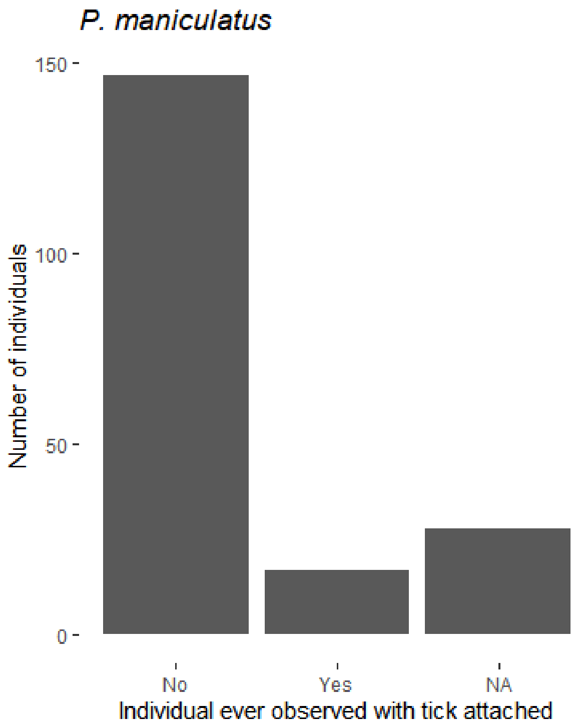

Supplement: Supplementary file 6 — Appendix S6. [file ECY-106-e4478-s001.pdf]
